# Evaluation of a commercial flatbed document scanner and radiographic film scanner for radiochromic EBT film dosimetry

**DOI:** 10.1120/jacmp.v11i2.3165

**Published:** 2010-04-19

**Authors:** Jason E. Matney, Brent C. Parker, Daniel W. Neck, Greg Henkelmann, Isaac I. Rosen

**Affiliations:** ^1^ Louisiana State University Department of Physics and Astronomy Baton Rouge LA; ^2^ Mary Bird Perkins Cancer Center Baton Rouge LA USA

**Keywords:** radiochromic film, Epson V700, Vidar Dosimetry Pro, EBT

## Abstract

The purpose of this study was to quantify the performance and assess the utility of two different types of scanners for radiochromic EBT film dosimetry: a commercial flatbed document scanner and a widely used radiographic film scanner. We evaluated the Epson Perfection V700 Photo flatbed scanner and the Vidar VXR Dosimetry Pro Advantage scanner as measurement devices for radiochromic EBT film. Measurements were made of scan orientation effects, response uniformity, and scanner noise. Scanners were tested using films irradiated with eight separate $3\times 3\;{\text{cm}}^{2}$ fields to doses ranging from 0.115–5.119 Gy. ImageJ and RIT software was used for analyzing the Epson and Vidar scans, respectively. For repeated scans of a single film, the measurements in each dose region were reproducible to within ±0.3% standard deviation (SD) with both scanners. Film‐to‐film variations for corresponding doses were measured to be within ±0.4% SD for both Epson scanner and Vidar scanners. Overall, the Epson scanner showed a 10% smaller range of pixel value compared to the Vidar scanner. Scanner noise was small: ±0.3% SD for the Epson and ±0.2% for the Vidar. Overall measurement uniformity for blank film in both systems was better than ±0.2%, provided that the leading and trailing 2 cm film edges were neglected in the Vidar system. In this region artifacts are attributed to the film rollers. Neither system demonstrated a clear measurement advantage. The Epson scanner is a relatively inexpensive method for analyzing radiochromic film, but there is a lack of commercially available software. For a clinic already using a Vidar scanner, applying it to radiochromic film is attractive because commercial software is available. However, care must be taken to avoid using the leading and trailing film edges.

PACS number: 87.55.Qr

## I. INTRODUCTION

Radiochromic film is gaining in popularity for use in x‐ray dosimetry. Unlike radiographic film, radiochromic film is self‐processing, eliminating the need for dedicated darkroom facilities and expensive wet chemical processing. High spatial resolution, minimal energy dependence,^(1)^ and near tissue equivalence^(2)^ make radiochromic film desirable for clinical film studies. EBT film is relatively insensitive to fluorescent room lighting. When using a phantom that requires cut film pieces, the ability to handle film in room light greatly simplifies the setup process.

Early types of radiochromic film were not quickly adopted in the clinic. Radiochromic films such as MD‐55 (International Specialty Products, Wayne, NJ) had low sensitivity and were suitable for measuring only very high doses (up to 100 Gy).^(3)^ ^,^ ^(4)^ The newest radio chromic film, GafChromic EBT (International Specialty Products, Wayne, NJ), is designed for the measurement of absorbed dose in the manufacturer specified range of 1–8 Gy and is more clinically useful than previous versions of radiochromic film.^(5)^ Radiochromic film has been shown to be an excellent tool for clinical film dosimetry provided that some issues such as film orientation during scanning, scanner selection, film handling and postirradiation scanning time are addressed.^(^ ^1^ ^,^ ^2^ ^,^ ^6^ ^–^ ^10^ ^)^

Currently, two types of scanners are available for use with radiochromic film dosimetry. Film scanners are commonly used in clinical settings to analyze radiographic films and can be used for radiochromic films as well. However, it has been suggested by the film manufacturer that for radiochromic film, a high quality flatbed document scanner may be sufficient and even possibly superior to traditionally used scanners.^(7)^ ^,^ ^(8)^ In the process of implementing radiochromic film dosimetry in our clinic, we evaluated a flatbed document scanner and a widely used film scanner. The performance of each scanner was measured in terms of constancy, uniformity, noise, and accuracy. Our results are compared to previous literature reports of other similar flatbed scanners.^(9)^ ^,^ ^(10)^ Reproducible methods of scanning were developed and verified for each scanner.

## II. MATERIALS AND METHODS

### A. Epson V700 Scanner system

**Figure 1:**
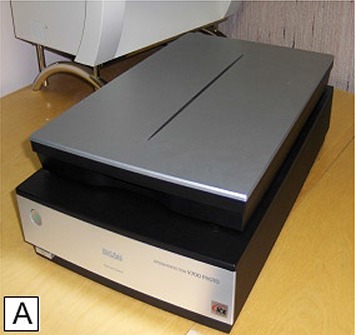
The Epson Perfection V700 Photo scanner (a)

An Epson Perfection V700 Photo scanner (Seiko Epson Corporation, Nagano, Japan), shown in Fig. 1(a), was purchased for testing as a radiochromic EBT film scanner. It is a flatbed document scanner designed for high‐quality photographic scanning. It is considered to be a replacement for the discontinued Epson Expression 1680 scanner, which has been widely used for radiochromic film dosimetry.^(^ ^6^ ^–^ ^8^ ^,^ ^11^ ^,^ ^12^ ^)^ The current cost of the V700 scanner is less than $1000.

The V700 utilizes a fluorescent light source with a broadband emission spectrum and a linear CCD array detector. For film dosimetry it is used in transmission mode, rather than reflection mode. The system has a maximum resolution of 6400 dots per inch (dpi), but this setting was not investigated. There is a large range of discrete pixel sizes that can be specified by the user. For this work, the resolution of all scans was set at 150 dpi (0.169 mm pixel size) to compromise between image resolution, file size and scan time. The maximum scan area is 21.6×29.7 cm.

In this scanner, the light source/detector system moves across the stationary film. Thus, it allows for repeat scanning of an individual film without moving it. Multiple scans of a single film can be averaged to reduce Type A measurement uncertainties. Scanned image data were stored in 48‐bit red‐green‐blue (RGB) tagged image file format (TIFF). The TIFF format is a lossless data‐compression technique and is amenable to processing by most image manipulation software. It also allows for extraction of the 16‐bit red channel from the 48‐bit RGB image. The EBT film manufacturer recommends using the red channel data for film analysis because its spectrum has an absorption peak at red light wavelengths.^(5)^ Scans were analyzed using the ImageJ (National Institutes of Health, USA) Version 1.40g (released April 18, 2008) software package.

The Epson scanner does not have an automatic warm‐up and self‐calibration routine. The user must acquire multiple warm‐up scans prior to acquiring dosimetry data. The user must also assume that the light source output is stable after the initial number of warm‐up scans. Paelinck et al.^(7)^ reported on the warm‐up characteristics – or short‐term drift – of the 1680 model Epson, but no one has reported on the short‐ or long‐term stability of the V700 Epson model.

### B. Vidar Scanner system

Our clinic currently uses the VXR Dosimetry PRO Advantage Film Digitizer (Vidar Systems Corporation, Hendon, Virginia), shown in Fig. 1(b), for all clinical and research radiographic film dosimetry. The Vidar manufacturer claims that its scanner is superior to Epson flatbed scanners for radiochromic film scanning.^(13)^ The Vidar has a fluorescent white light source with a spectral distribution ranging from 250 to 750 nm and a linear CCD system for measuring transmitted light. The pixel size can be set to 0.356, 0.178 or 0.089 mm. It produces a 16‐bit grayscale image using the entire spectrum of the scanner light source. It can be used with the established commercial RIT V5.0 (Radiological Imaging Technology, Colorado Springs, CO) software for image analysis. This scanner currently costs over $10,000 including licensing fees and the necessary analysis software.

The Vidar scanner can accept films up to 35.5 cm wide. Film is transported past the measurement apparatus, so film must be manually loaded into the scanner before each scan. Because the film moves through the Vidar scanner, the physical movement of the film does not allow for repeat scanning of the same film in exactly the same position. The film must be replaced by hand in order to rescan in the Vidar system. Therefore, the user is unable to average a number of repeat scans to reduce the uncertainty on a film scan. The Vidar includes a forced self‐calibration routine to ensure that the light source is sufficiently warmed up and stable. It automatically repeats a shorter calibration routine to ensure the light source is still sufficiently stable before every film is scanned.

### C. Test films

**Figure 2:**
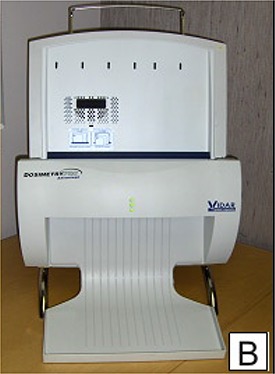
The VXR Dosimetry PRO Advantage Film Digitizer (b).

Test films were created using an MLC defined eight‐box radiographic film calibration technique described by Childress et al.^(14)^ Films were irradiated with a range of doses from 0.115 to 5.119 Gy. Films were irradiated using a 6 MV beam from a Varian EX linac. They were positioned perpendicular to the beam at 100 cm SAD and 10 cm depth in solid water, with 10 cm of solid water below the film to provide backscatter. Radiation was delivered using a predefined multileaf collimator sequence that created two columns of four $3\times 3\;{\text{cm}}^{2}$ regions. Following the technique of Childress et al., an ion chamber (Model A1SL Exradin Miniature Shonka Thimble Chamber) was used to measure the absolute dose at the centers of each of the eight dose regions and at a low dose region in the center of the fields that received only scatter radiation. The ion chamber used has a collecting volume of 0.057 cm^3^ and a 6.4 mm diameter. The time‐integrated ion chamber measurement for each dose included primary, scatter, and leakage dose contributions from all eight fields delivered to the film. Figure 2 shows the eight visible dose regions (labeled 1–8), and the low dose region (labeled as region 0). Dose regions 0–8 received doses of 0.115, 0.673, 1.378, 1.998, 2.592, 3.204, 3.926, 4.538, and 5.119 Gy, respectively. These doses cover the majority of the manufacturer‐specified dynamic range for EBT film (up to 8 Gy).

All five films were irradiated and processed in the same way. The film‐to‐film variation test was done with a group of films all from the same batch and all irradiated in the same session. Film exposure to room lighting was minimized and approximately equal for all films. The films were labeled and stored for a period of 24 hours post‐irradiation before scanning. This time interval was chosen to ensure that all polymer changes within the film due to the irradiation had been completed. The manufacturer recommends waiting at least two hours post‐irradiation before scanning. As per manufacturer suggestion, the films were stored together before and after irradiation to maintain the same thermal histories.

### D. Film scanning procedures

The experiment films were uncut sheets of EBT film $\left(20.3\times 25.4\;{\text{cm}}^{2}\right)$ and smaller than the maximum scanner bed size on both scanners. Therefore, experiment films were positioned in the center region of both scanners for scanning. Due to scanner differences, the same pixel size could not be used by each scanner. Pixel size was chosen to be similar in both scanners: 178 μm for the Vidar scanner and 169 μm (150 dpi) for the Epson scanner. This was done in order to balance a small pixel size with an increasing scan time and overall size of the image files generated. The small difference in pixel size was ignored.

Because the Epson scanner has no warm‐up or calibration procedure, it was allowed to warm up for at least 30 minutes prior to each scanning session. Prior to data collection, ten scans were performed with no film in place to allow stabilization of the light source. Experiment films were positioned in the center of the scanner bed. All image enhancement features were turned off in the Epson control software. The 16‐bit red channel was extracted from the RGB image and measured and analyzed. As previously stated, ImageJ software was used to analyze all Epson scans.

For all Vidar scans, films were scanned in the portrait orientation. The RIT software was used for film measurements and analysis. No image processing was performed. The EBT manufacturer recommends, and will provide, a clear polyester sleeve to secure film pieces for transport through the Vidar system. We found these sleeves to be unacceptable due to circular Newton ring artifacts caused by interference patterns in the light reflected through different thicknesses of the air layer between the sleeve surfaces. These rings were clearly visible on the resulting film scan images. The film sleeve technique was not used for this work.

### E. Scanner testing

For each test film, we measured the mean pixel value in the central $5\times 5\;{\text{mm}}^{2}$ area of each of the eight irradiated regions. This mean signal is referred to as the “film response” and represents the measurement value corresponding to delivered dose. This measurement area was large enough to give good statistics and still small enough to avoid penumbra effects near the edges of the irradiation squares. For the region of low dose, a $5\times 5\;{\text{mm}}^{2}$ area was chosen in the center of the film to represent region 0, as shown in Fig. 2.

#### E.1 Scanner consistency

To test the consistency of each scanner, a single test film was scanned 10 times in succession on each system. For each of the nine dose regions, we measured the mean film response and the standard deviation of the film response. This test also incorporates any error in selecting the $5\times 5\;{\text{mm}}^{2}$ region of interest for measurement. Because the same film was used for all the scans, the standard deviation is a measure of the scanner consistency and selection of the location of the measurement box.

#### E.2 Film‐to‐film variation

The variation among films from a single batch was measured to evaluate the sensitivity of the scanner to film variations and to determine if a single calibration curve could be applied to all films from one batch. Five films from a single batch were irradiated sequentially in one session to minimize linear accelerator output variations. Therefore, each film received the same dose in each of the corresponding dose regions. For each of the nine dose regions over the five films, we measured the mean film response and the standard deviation of the film response.

#### E.3 Film orientation effects

The optical density of EBT film has been shown to change substantially depending on the polarization of the analyzing scanner light relative to the orientation of the film.^(15)^ Zeidan et al. reported a decrease in optical density of 50% at 50 cGy and 25% at 200 cGy with a 90° rotation of the film in an Epson 1680 scanner. This effect was attributed to the alignment of the radiochromic film polymer chains that form after irradiation.^(16)^ Because the scanners have different light source spectra and detector properties, we measured the film orientation effect in both scanning systems.

A single test film was scanned in both portrait and landscape orientations. Portrait orientation was defined as the long axis of the film being perpendicular to the linear scanner light source; landscape orientation was defined as the long axis of the film being parallel to the linear light source. The film response in corresponding dose regions in both orientations were compared in terms of percent difference. The portrait orientation was selected as the reference scan orientation because all previous films were scanned in this orientation.

#### E.4 Scanner uniformity

A single sheet of unirradiated radiochromic film was scanned in portrait orientation using both systems. Horizontal and vertical profiles were taken through the center of the film, and the mean and standard deviation of the signal were calculated across the profile. This test assumed that the unirradiated film was of uniform optical density over the entire sheet. The horizontal profile measured the uniformity of the light and detector system combination across its scanning range. The vertical profile across the films is recorded by the same light and detector segments throughout the entire scan. The vertical profile of a blank film measures the uniformity of response of a single detector segment throughout a scan and the stability of the light source opposite that particular detector element.

#### E.5 Film/scanner noise

A single test film from the five used in Section E.1 above (scanner consistency) was used to measurement overall noise. This noise measurement incorporates scanner, film, and irradiation variations, and gives the precision of pixel value measurement as a function of delivered dose. The measurement noise of the scanner/film system was taken to be the measured standard deviation of all the pixels in the central $5\times 5\;{\text{mm}}^{2}$ of each dose region. This measurement was performed for both scanners.

## III. RESULTS

### A. Scanner consistency

The scanner consistency results for both systems are shown in Table 1. The Table shows the average of the 10 measured film responses (μ1E and μ1V) and the standard deviation of the 10 mean values (σ1E and σ1V) expressed as a percentage of the mean at each dose. Epson data is denoted by a subscript E and Vidar data is denoted by a subscript V. The maximum standard deviation of the mean for the Epson and Vidar scanner was less than 0.2% and 0.3%, respectively, for repeat scanning and measurement of the same film.

**Table 1:** Consistency of a single test film scanned ten times; reported by the mean (μ1E and μ1V) and standard deviation (σ1E and σ1V) of the distribution of the means. Film‐to‐film response over five test film given by the mean (μ5E and μ5V) and standard deviation (σ5E and σ5V) measured using the two scanners. Epson values denoted by subscript E; Vidar values denoted with subscript V.

*Epson*					*Vidar*			
*Dose (Gy)*	μ1E	σ1E *(%)*	μ5E	σ5E *(%)*	μ1V	σ1V *(%)*	μ5V	σ5V *(%)*
0.115	47592	0.2	46456	0.1	45062	0.1	45701	0.2
0.673	37719	0.2	37423	0.3	34422	0.1	36699	0.4
1.378	31217	0.2	31103	0.1	28536	0.2	30227	0.3
1.998	27069	0.2	26862	0.2	24602	0.2	26000	0.2
2.592	24260	0.1	24097	0.4	22021	0.3	23219	0.3
3.204	21682	0.2	21773	0.3	20097	0.1	21057	0.3
3.926	19806	0.1	19991	0.4	18511	0.1	19101	0.4
4.538	18501	0.1	18462	0.4	17292	0.1	17597	0.1
5.119	17435	0.1	17335	0.2	16249	0.1	16434	0.1

### B. Film‐to‐film variation

The mean (μ5E and μ5V) and standard deviation (σ5E and σ5V) of the five experimental films from the same batch are expressed as a percentage of the mean in Table 1. For both scanners, the largest standard deviation of the film response is observed to be 0.4% of the mean. This is approximately the same as the observed standard deviation when repeatedly scanning the same film multiple times as reported in Section A above (scanner consistency).

### C. Film orientation effect

The effects of film orientation are reported in Table 2. The two columns of Table 2 show the percent difference from portrait orientation to landscape orientations, given by:

(1) $$\%diff=\frac{\mu_{Land}-\mu_{Portrait}}{\mu_{Portrait}}$$

where $\mu_{\text{Land}}$ and $\mu_{\text{Portrait}}$ are the mean film response at each dose region in landscape and portrait orientation, respectively.

**Table 2:** Percent difference of film response between portrait and landscape film orientation measured by Epson and Vidar scanners over a range of doses.

*Dose (Gy)*	*Epson*	*Vidar*
0.115	6%	1%
0.673	9%	1%
1.378	11%	2%
1.998	13%	3%
2.592	13%	3%
3.204	16%	4%
3.926	17%	5%
4.538	17%	5%
5.119	16%	4%

The tests show that the film orientation affects the results from both scanners, but the effect is much larger for the Epson scanner. The largest change measured with the Epson scanner was 17%, while the largest change observed with the Vidar was 5%. The larger susceptibility of the Epson to orientation effects may be due to using only the red light portion of the Epson's RGB image, which cannot be done on Vidar produced images.

### D. Scanner uniformity

**Figure 3:**
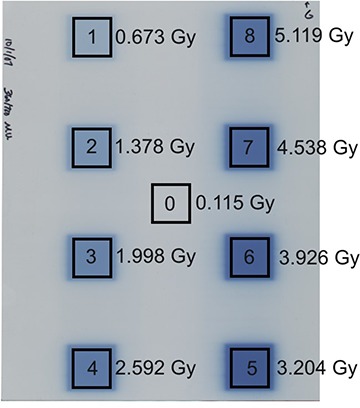
Radiochromic EBT test film irradiated using the eight‐box calibration technique. The eight irradiated regions (1–8), the central low dose scatter region (0), and corresponding doses are shown.

Uniformity of scanner response was measured by evaluating horizontal and vertical profiles taken through the central axis of an unirradiated film, shown in Fig. 3. The Vidar scanner measured higher pixel values than the Epson scanner. This is due to inherent differences in the light sources, detectors, and analog‐to‐digital converters. The magnitude of Epson data is also affected by using only the red light channel rather than the total image value.

In the vertical profile, the measurement shows the response of a single central detector along the film. If we consider this detector to be stable over the scan duration, the variation measured in the vertical profile gives us a measure of the nonuniformity across the film. Nonuniformities are seen in the first and last two centimeters of the Vidar vertical profile that are not observed in the Epson vertical profile. These nonuniformities are produced only by the Vidar scanner images, and are not inherent in the film. This is attributed to the Vidar film transport roller mechanism, as reported by Wilcox, et al.^(17)^ Ignoring the leading and trailing regions (~2 cm) of film, the Vidar vertical profile has a mean value of 50653 and a standard deviation of 0.3% of the mean. The Epson scanner vertical profile has a mean value of 49060 and a standard deviation of 0.3% of the mean. Thus, when the roller artifacts are ignored in the Vidar system, both systems have similar vertical response uniformity. This confirms the film uniformity as per manufacturer's specifications. Also, the output of the light source and detector response remain constant during the course of a scan, as expected.

**Figure 4:**
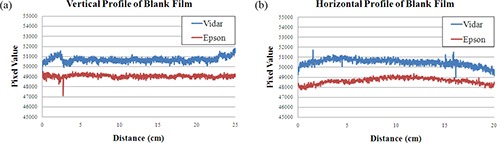
Vertical profiles of a blank film as measured by the Epson and Vidar scanner systems (a); horizontal profiles of a blank film as measured by the Epson and Vidar scanner systems (b).

The horizontal profile gives a measure of variation across all of the detectors that the film passed through during measurement, including film nonuniformity. In the horizontal direction, no artifacts were seen in either system. The Vidar scan has a mean value of 50464 with a standard deviation of 0.7%. The Epson scan is similar with a mean of 48646 and standard deviation of 0.6%.

The film manufacturer quotes that the film has a uniformity variation of 1.5%. Thus, both scanners measure a uniformity for a blank film that is within the quoted film uniformity tolerances. From this test, both scanners appear to be uniform to within the uniformity of the film itself, given the leading and trailing film edges are ignored in the Vidar scanner. These numbers suggest that the actual film uniformity may be better than the manufacturer specification.

### E. Film/scanner noise

Table 3 shows, for each scanner, the average standard deviation over five films at each dose level measured. The standard deviation of each region is taken to be the overall measurement noise, and includes scanner, film, and irradiation variations. The Table gives standard deviation (σ) expressed as a percentage of the mean. For the Epson and Vidar systems, the average standard deviation was less than 0.3% and 0.2%, respectively, for all doses.

**Table 3:** Average standard deviation of pixel value within the $5\times 5\;{\text{mm}}^{2}$ measurement region for the Epson and Vidar scanner systems.

*Dose (Gy)*	*Avg. σ Epsom*	*Avg. σ Vidar*
0	0.1%	0.2%
0.115	0.1%	0.2%
0.673	0.2%	0.2%
1.378	0.2%	0.2%
1.998	0.2%	0.2%
2.592	0.3%	0.2%
3.204	0.3%	0.2%
3.926	0.3%	0.2%
4.538	0.3%	0.2%
5.119	0.1%	0.2%

## IV. DISCUSSION

Both scanners showed similar performances in terms of measurement consistency, response uniformity, and overall measurement noise. The Epson slightly outperformed the Vidar scanner in scanner noise and scanner uniformity. We found that film‐to‐film variations within a single batch of film were very low, with standard deviations of less than 0.4% for the Epson and Vidar scanner. The variation within a repeat scan of a film yielded a standard deviation less than 0.3% of the mean.

Film orientation was found to have a significant impact measurement results for both systems. The effect was much larger for the Epson scanner using the red light signal. Consequently, care must be taken to maintain a consistent orientation when processing all films for an experimental measurement.

A nonuniformity was observed with the Vidar scanner on the leading and trailing edges of a scanned film. This effect has been reported by Wilcox, et al.^(17)^ and was confirmed in our testing. The leading and trailing edge of the film profile perpendicular to the light source measured a 3% increase in pixel value. The artifacts can be avoided by ignoring the leading and trailing section of film in a film scan, or by affixing the piece of experiment film to a larger film guide for transportation through the Vidar scanner.

One source of uncertainty with the Epson scanner is that there no self‐calibration of light source or warm‐up tests exist. Paelinck reported issues with the Epson 1680 scanner warm‐up,^(7)^ but this work found no such issues with the V700 scanner. The user must simply take several preview scans with the Epson scanner to adequately warm up the scanner. In this testing, no drift was observed during repeated film scanning. In contrast, the Vidar scanner performs a self‐calibration at the start of every run of film analysis. It would be ideal if a similar warm‐up and calibration interface could be designed for use with Epson scanners.

Both film scanners proved to be reliable and accurate for film dosimetry. The film manufacturer does recommend the FilmQA software (3cognition, Wayne, NJ) for scanning EBT film using an Epson scanner. However, this work did not warrant the purchase of the FilmQA program. Attempts to utilize the RIT software system with the Epson scanner were not successful. Therefore, for clinical use, we decided to use the Vidar scanner. The Vidar scanner and the commercial RIT 5.0 software system have proven sufficiently useful to warrant not developing or purchasing software for using the Epson scanner. If an institution already has a working Vidar scanner system, we cannot see a clear reason to adopt an Epson scanner system for EBT radiochromic film. However, if an institution does not have a radiographic film scanner similar to the Vidar, the Epson scanner may be an attractive alternate to purchasing a Vidar scanner specifically for radiochromic film dosimetry.

## V. CONCLUSIONS

We tested two potential radiochromic film scanners, the Epson Perfection V700 Photo flatbed scanner and the Vidar VXR Dosimetry PRO Advantage Film Digitizer. The scanners were tested for film constancy, film‐to‐film variation, film orientation effects, scanner uniformity, and scanner noise. We found that both scanners gave similar performance over the range of tests. We conclude that the selection of scanner is up to the individual user, based on the current hardware and software availability. The Vidar scanner system is already well‐established for radiographic film dosimetry and, thus, may be an attractive option for users with an existing system. However, the Epson scanner is a cost‐effective scanner that is also capable of scanning radiochromic film. Care must be taken when implementing a new film dosimetry system, especially one using radiochromic EBT film which has several key differences from conventional radiographic film systems. The user should fully test and validate any new scanner used for film dosimetry.
